# Correlation between Glasgow coma scale and brain CT-scan findings in traumatic patients

**Published:** 2012-11

**Authors:** Nazanin Farshchian, Firoozeh Farshchian, Mansour Rezaei

**Affiliations:** ^*a*^Kermanshah University of Medical Sciences, Kermanshah, Iran.; ^*b*^Kermanshah University of Medical Sciences, Emam Reza Hospital, Kermanshah, Iran.

## Abstract

**Background:**

Traumatic brain injury (TBI) is a common problem caused by multiple traumas. The TBI patients are clinically evaluated with Glasgow Coma Scale (GCS) as well as imaging methods including computed tomography (CT) scan and magnetic resonance imaging (MRI). According to the GCS scores, patient are divided into three groups: mild (13-15), moderate (9-12) and severe (3-8). This study aims to investigate correlation between GCS and CT-scan findings.

**Methods:**

In this cross-sectional study, a total of 432 patients were evaluated with both brain CT-scan and GCS in their first 24 hours post trauma in the Emam Reza hospital (Kermanshah, Iran) during 2011.

**Results:**

385 patients (89.12%) were male. Average age of patients was 25 ± 5 (SD) years. The most common causes of TBI were car accidents (72.9%), violence (20.8%) and fallings (6.27%). 70.13% of patients had mild, 7.8% had moderate and 22% had sever TBI. Of all patients, 79.86% cases had positive brain CT, of which 53.47% had subgaleal hematoma and 28.74% extra-axial hematoma as the most common indications.

**Conclusions:**

Findings of this study showed that more than three positive indications including extra-axial, hematoma, subarachnoid hemorrhage, and hemorrhagic contusion are associated with low GCS scores and moderate or severe TBI.

**Keywords:**

Brain trauma, Coma, Brain CT-scan, Glasgow Coma Scale


					Volume 4, Suppl. 1 Nov 2012
Publisher: Kermanshah University of Medical Sciences
URL: http://www.jivresearch.org
Copyright:
Congress of Iranian Neurosurgeons, 14-16 November 2012, Kermanshah, Iran
Paper No. 44
Correlation between Glasgow coma scale and brain CT-scan
findings in traumatic patients
Nazanin Farshchian a,*
, Firoozeh Farshchian b
, Mansour Rezaei a
a
Kermanshah University of Medical Sciences, Kermanshah, Iran.
b
Kermanshah University of Medical Sciences, Emam Reza Hospital, Kermanshah, Iran.
Abstract:
Background: Traumatic brain injury (TBI) is a common problem caused by multiple traumas. The TBI patients are
clinically evaluated with Glasgow Coma Scale (GCS) as well as imaging methods including computed tomography
(CT) scan and magnetic resonance imaging (MRI). According to the GCS scores, patient are divided into three groups:
mild (13-15), moderate (9-12) and severe (3-8). This study aims to investigate correlation between GCS and CT-
scan findings.
Methods: In this cross-sectional study, a total of 432 patients were evaluated with both brain CT-scan and GCS in
their first 24 hours post trauma in the Emam Reza hospital (Kermanshah, Iran) during 2011.
Results: 385 patients (89.12%) were male. Average age of patients was 25  5 (SD) years. The most common
causes of TBI were car accidents (72.9%), violence (20.8%) and fallings (6.27%). 70.13% of patients had mild,
7.8% had moderate and 22% had sever TBI. Of all patients, 79.86% cases had positive brain CT, of which 53.47%
had subgaleal hematoma and 28.74% extra-axial hematoma as the most common indications.
Conclusions: Findings of this study showed that more than three positive indications including extra-axial, hematoma,
subarachnoid hemorrhage, and hemorrhagic contusion are associated with low GCS scores and moderate or severe
TBI.
Keywords:
Brain trauma, Coma, Brain CT-scan, Glasgow Coma Scale
* Corresponding Author at:
Nazanin Farshchian: Radiology Specialist, Assistant Professor, Kermanshah University of Medical Sciences, Kermanshah, Iran. Mob: 09123757729,
Email: n_farshchian@yahoo.com, (Farshchian N.).

				

